# Evaluating lexical similarity and modeling discrepancies in the procedure hierarchy of SNOMED CT

**DOI:** 10.1186/s12911-018-0673-z

**Published:** 2018-12-12

**Authors:** Ankur Agrawal

**Affiliations:** Department of Computer Science, Manhattan College, New York, NY USA

**Keywords:** SNOMED CT, Quality assurance, Auditing, Contextual, Lexical analysis, Similarity sets

## Abstract

**Background:**

SNOMED CT is a standardized and comprehensive clinical terminology that is used in Electronic Health Records to capture, store and access clinical data of patients. Studies have, however, shown that there are inconsistencies inherent in the modeling of concepts in SNOMED CT that can have an impact on its usage to record clinical data and in clinical decision-making tools.

**Methods:**

An effective lexical approach to identifying inconsistencies with high likelihood in the structural modeling of the concepts of SNOMED CT is discussed and assessed. The approach uses the two or more concepts in the context of their lexical similarity to compare their modeling in order to identify inconsistencies. A sample of 50 sets is randomly picked from the Procedure hierarchy of SNOMED CT and evaluated for inconsistencies.

**Results:**

Of the 50 randomly picked sets, 58% are found to exhibit one or more concepts with inconsistencies. In terms of concepts, 29% of the 146 concepts are found to exhibit one or more inconsistencies.

**Conclusions:**

The assessment of the sample concepts shows that SNOMED CT is not free from inconsistencies which may affect its use in clinical care and decision support systems. The proposed methodology is found to be effective in identifying areas of SNOMED CT that may be in need of quality assessment.

## Background

SNOMED CT [1] is a comprehensive and standardized clinical reference terminology that can be used in electronic health records (EHRs) to facilitate the storage and transmission of patient data in a consistent and reliable way. Since the clinical coding is standardized, SNOMED CT allows automatic interpretation of these codes thus enabling clinical decision making. SNOMED CT is also one of the requirements to be used with EHRs to be eligible for the meaningful use incentive program [2–4] which was introduced by the Health Information Technology for Economic and Clinical Health Act (HITECH) [5] to promote the adoption and meaningful use of health information technology.

The origin of SNOMED CT dates to over 50 years [6]. Systematized Nomenclature of Pathology (SNOP) was published by the College of American Pathologists in 1965 which was later expanded in 1975 to form Systematized Nomenclature of Medicine (SNOMED). SNOMED II was released in 1979 followed by SNOMED International, or SNOMED 3.0 in 1993. This was followed by SNOMED RT in 2000 which was merged with Clinical Terms Version 3 (CTV3) to form the first version of SNOMED CT in 2002. Presently, SNOMED CT is being used by health care providers in over fifty countries.

SNOMED CT consists of concepts with unique meaning that are organized into 19 hierarchies. Examples of hierarchies include procedure, clinical finding, specimen and body structure. All but the root concept (SNOMED CT Concept (SNOMED RT + CTV3)) has one or more parent concepts and zero or more child concepts which form the hierarchical (is a) relationships. The concepts may also have zero or more attribute relationships. These attributes extend between concepts of multiple hierarchies. Some concepts may have their attributes grouped into relationship groups to add clarity to concept definitions. The concepts may also have multiple synonyms associated with them. All concepts have a fully specified name that is used to unambiguously identify a concept and a preferred term that is commonly used by clinicians to identify a concept.

The usage and implementation of SNOMED CT has been discussed in several studies. The need for a global clinical language and how SNOMED CT fits in is discussed in [7]. Lee et al. have listed several implementations of SNOMED CT in [8] based on their survey of individuals and companies that have been using SNOMED CT for clinical purposes. The authors found SNOMED CT as being used to encode various clinical information including patient summary and medical history, signs and symptoms, problems and complaints and encompassing different domains such as intensive care, primary care and specialist care among others.

The content of SNOMED CT, however, is not free from inconsistencies. Several published studies have conducted review of the content of SNOMED CT and found issues with the coverage and the content of the terminology. In [9], Rector et al. discuss various modeling problems affecting the use of SNOMED CT in practical applications. Some of the modeling problems they discuss in SNOMED CT January 2010 release include “Myocardial infarction” not classified as “Ischemic heart disease” and “Injuries of the dorsalis pedis artery” being inferred as a kind of “Injury of the abdomen” and “Injury to the pelvis”.

Quality assurance plays an integral role in maintaining the quality of a terminology [10]. Several studies have been published in the past 10 years that deal with the quality assurance of SNOMED CT and assess its completeness and accuracy. In [11], Elhanan et al. presented the results of a survey of the direct users of SNOMED CT and their desire to improve the consistency, quality and completeness of the content of SNOMED CT. In [12], Zhu et al. presented a literature review of the auditing methods applied to various biomedical terminologies including SNOMED CT. A critical review of the structure of SNOMED CT and recommendations were presented in [13].

Owing to the comprehensiveness of SNOMED CT and limited resources available for auditing, it is imperative to have some algorithmic way of identifying problematic areas of SNOMED CT that has high likelihood of exhibiting inconsistencies thus requiring greater scrutiny. This study presents a lexical technique to identify inconsistencies in hierarchical relationships, attributes and relationship groups. In [14–16], a contextual methodology was introduced that used a lexical approach to identify areas in SNOMED CT that are candidate for manual auditing. The current study is an extension of this methodology and aims at evaluating the concepts in the Procedure hierarchy of SNOMED CT. The study modifies the algorithm as described in the Methods section which results in more concepts being eligible to be analyzed by the algorithm and to identify any inherent consistencies in their structural modeling.

## Method

The methodology presented in this study is based on the assumption that concepts with a lexically similar description are expected to be modeled in a similar fashion. That is, the two concepts that are lexically similar should have same or similar hierarchical relationships, attributes and relationship groups. For instance, consider the two concepts in Table 1. These two concepts are lexically similar as their fully specified names only differ by one word – tarsometatarsal vs. midtarsal.

**Table 1:** Example of two lexically similar concepts

Tarsometatarsal arthrodesis, transverse, with osteotomy as for flatfoot correction (procedure)	
Midtarsal arthrodesis, transverse, with osteotomy as for flatfoot correction (procedure)	

**Fig. 1:**
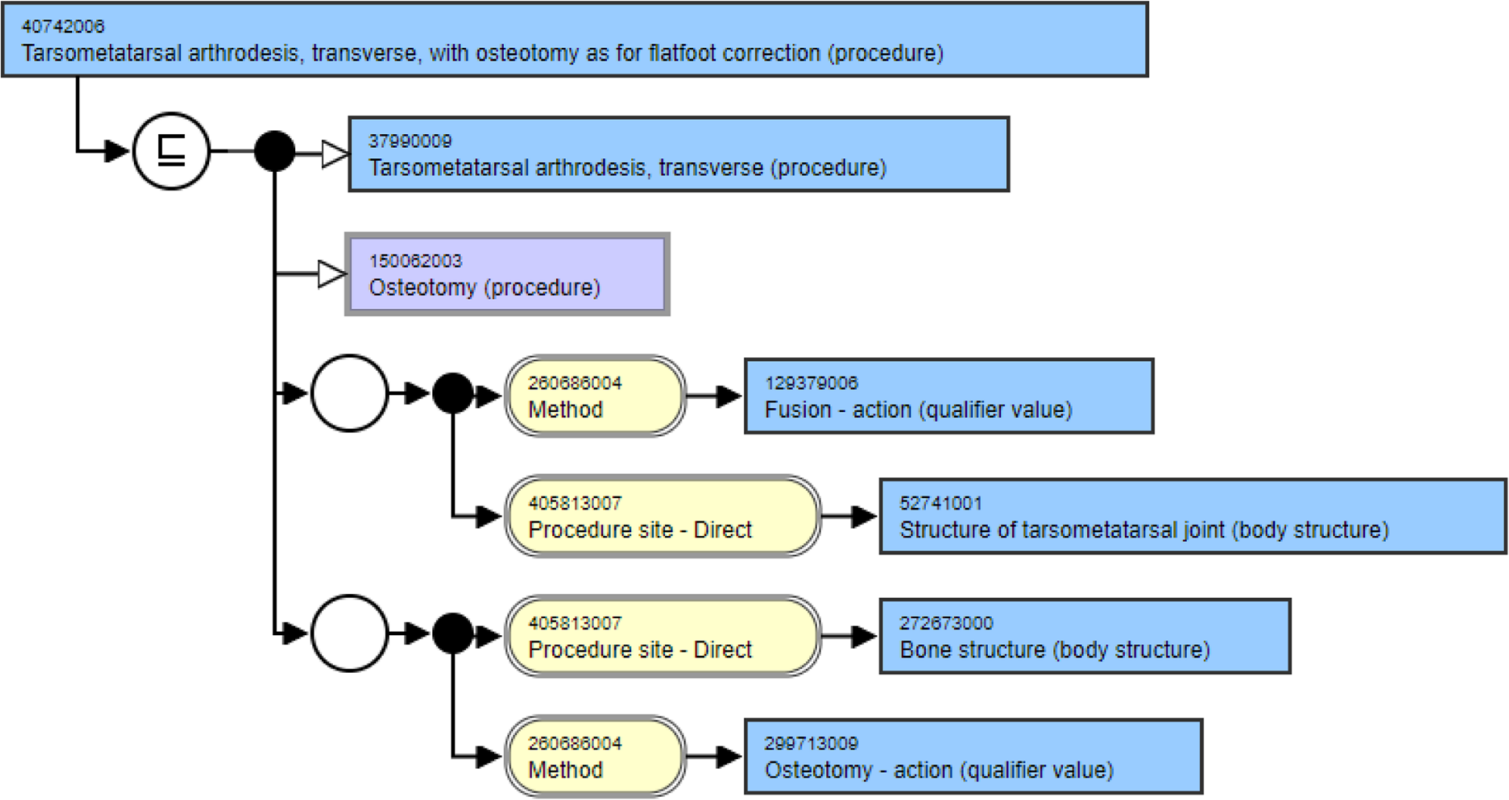
Structural modeling of concept with Concept ID 40742006

**Fig. 2:**
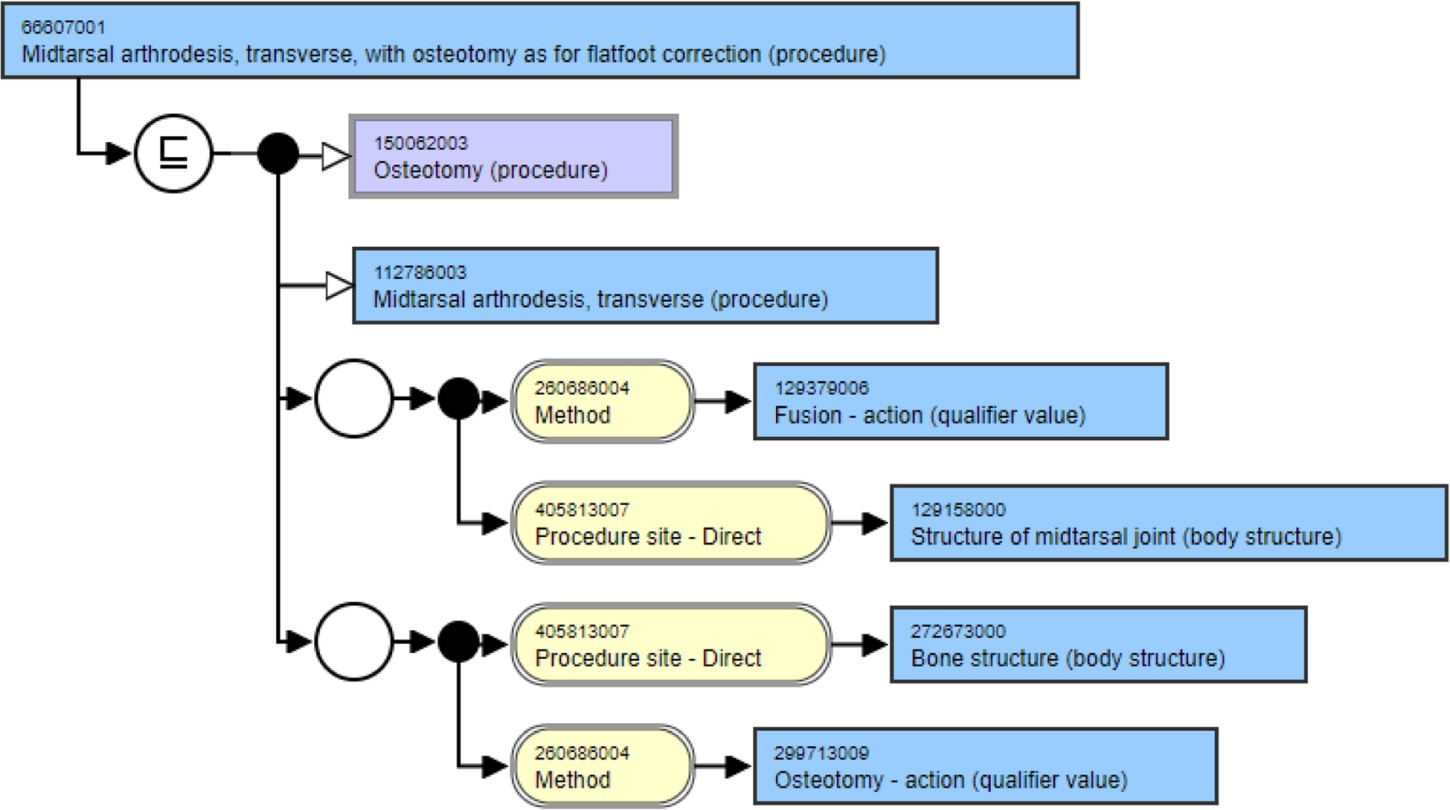
Structural modeling of concept with Concept ID 66607001

The structural modeling of the two concepts from Table 1 is shown in Fig. 1 and Fig. 2 that have been downloaded from SNOMED International’s SNOMED CT browser [17]. A look at the structure of these two concepts reveal that they are modeled in a similar fashion. Both these concepts have two parents, one being the same (Osteotomy) whereas the other being lexically similar (differing by just a word - Tarsometatarsal arthrodesis, transverse vs. Midtarsal arthrodesis, transverse). Both the concepts have two relationship groups with two attributes in each group. Both the attributes in the corresponding relationship groups are the same (Method and Procedure site - Direct). Three of the four attribute target values are the same (Fusion – action, Osteotomy – action and Bone structure) with the fourth one being similar lexically (Structure of tarsometatarsal joint vs. Structure of midtarsal joint). This is the kind of similarity in the structural modeling of concepts that is expected from lexically similar concepts.

The methodology described in this study builds on this observation to identify inconsistencies in the modeling of similarly worded concepts. For each concept in Procedure hierarchy of SNOMED CT, their descriptive terms are extracted which includes the fully specified name and synonyms. Each descriptive term of each concept is then converted into a list of words by breaking down the terms into individual words. Stop words such as a, an, the, etc. are removed from these lists.

Each list of words formed from the descriptive terms of a concept is then compared with that of every other concept in the hierarchy. Only those concepts with five or more words in their fully specified terms after removing the stop words are considered. If two or more concepts differ from each other by just one word in any of their descriptive terms, they are grouped together to form a set of similar concepts called similarity sets. The concepts in Table 1 form a similarity set and so do the concepts in Table 2.

**Table 2:** Similarity set with two concepts

Drainage of lesion of pelvis using computed tomography guidance (procedure)	
Computer tomography guided drainage of pancreatic lesion (procedure)	

In Table 2, while the fully specified names of the two concepts differ by three words – [pelvis, computed, guidance] as against [computer, guided, pancreatic], their synonyms differ by only one word – pelvis vs. pancreatic, as can be seen in the Table 3. These two concepts thus form a similarity set based on lexically similar synonyms.

**Table 3:** Similar synonyms of concepts from Table 2

CT guided drainage of lesion of pelvis	
CT guided drainage of pancreatic lesion	

The position of the matching and differing words between the two concepts is not considered in a similarity set. This enables the methodology to consider more concepts than it would by taking into consideration the position of the words. Consider the two concepts forming a similarity set as shown in Table 4. While the position of the differing word - surgical vs. non-surgical - is the same, the position of the matching words [biopsy, gastrointestinal, tract] are different within the fully specified names of the two concepts. Also, in the example in Table 3, the differing words, pelvis and pancreatic, are at different positions within the synonyms which is acceptable in the formation of a similarity set.

**Table 4:** Similarity set with concepts having similar fully specified names

Surgical biopsy of gastrointestinal tract (procedure)	
Non-surgical gastrointestinal tract biopsy (procedure)	

There are two distinct kinds of similarity sets generated. The first kind (Same_Sets) is the one where all concepts in the set have the same number of hierarchical relationships, attributes and role groups. This is what one can expect from lexically similar concepts as shown in Fig. 1 and Fig. 2. The second kind (Diff_Sets) is the one where at least one concept in the set differ from the rest in the number of hierarchical relationships, attributes and/or role groups. These are the sets that are regarded as inconsistent and need further scrutiny.

Similarity sets are generated for each concept in the Procedure hierarchy of SNOMED CT using January 2018 release. Care is taken to avoid formation of duplicate sets. A random sample of 50 sets of the second kind (Diff_Sets) is picked which is then evaluated for inconsistencies by a single auditor. For this study, the size of the sets in the random sample is limited to a maximum of four concepts, that is, only those sets that have two, three or four concepts are considered for the sample. The auditor looked for three kinds of inconsistencies – hierarchical (missing and/or incorrect), attribute-related (missing and/or incorrect attribute, incorrect target value) and relationship group-related (incorrect and/or missing).

## Results

The Procedure hierarchy consists of 57,805 active concepts. A total of 13,202 similarity sets are generated by the algorithm utilizing 20,658 of the concepts from the Procedure hierarchy. As such the methodology accounts for 36% of the concepts from the hierarchy. A total of 73% of the 13,202 similarity sets are Diff_Sets while the rest 27% are Same_Sets.

The randomly selected sample from Diff_Sets consists of 50 sets of which 29 (58%) were found to have one or more inconsistent concepts. The 50 sets consisted of 146 concepts of which 42 (29%) were found to exhibit one or more kinds of inconsistencies as shown in Table 5.

**Table 5:** Types of inconsistencies

Type of inconsistency	**#**	%
Concepts with hierarchical inconsistencies	32	22
Concepts with attributes and target values related inconsistencies	28	19
Concepts with relationship groups related inconsistencies	9	6

Table 6 and Table 7 summarize the different kinds of sets and inconsistencies within them in the random sample. There were 41 sets (also called Diff-Par sets) with at least one concept in the set having different number of parents than the rest of the concepts in the set. Of these 41 sets, 24 sets were found to exhibit one or more inconsistent concepts. These 41 sets had a total of 124 concepts of which 37 were found to exhibit one or more inconsistencies. There were 27 sets (also called Diff-Rel sets) with at least one concept in the set having different number of attributes than the rest of the concepts in the set. Of these 27 sets, 17 sets were found to exhibit one or more inconsistent concepts. These 27 sets had a total of 78 concepts of which 27 were found to exhibit one or more inconsistencies. There were 13 sets (Diff-Grp sets) with at least one concept in the set having different number of attributes than the rest of the concepts in the set. Of these 13 sets, 8 sets were found to exhibit one or more inconsistent concepts. These 13 sets had a total of 40 concepts of which 14 were found to exhibit one or more inconsistencies.

**Table 6:** Summary of inconsistent sets in different set types

	Total Sets	Inconsistent Sets	
		#	%
Diff-Par Sets	41	24	59
Diff-Rel Sets	27	17	63
Diff-Grp Sets	13	8	62
Overall	50	29	58

**Table 7:** Summary of inconsistent concepts in different set types

	Total Concepts	Inconsistent Concepts	
		#	%
Diff-Par Sets	124	37	30
Diff-Rel Sets	78	27	35
Diff-Grp Sets	40	14	35
Overall	146	42	29

## Discussion

The methodology described in this study uses a contextual auditing technique where the modeling of a concept is considered in the context of the modeling of a lexically similar concept. An important advantage of this technique is that it helps identify inconsistencies which would otherwise be difficult to uncover manually by looking at a concept on its own.

The use of synonyms in addition to fully specified names made it possible to consider concepts in similarity sets and uncover inconsistencies which would not have been possible in some of the previous studies [14–16, 18] that did not use synonyms to form similarity sets. There were additional 655 sets formed and 993 additional concepts were considered from the Procedure hierarchy because of the inclusion of synonyms in the methodology.

**Fig. 3:**
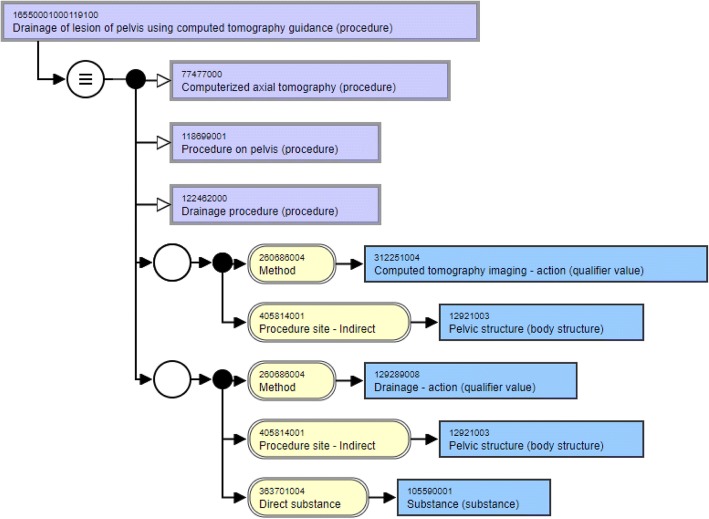
Structural modeling of concept with Concept ID 16550001000119100

**Fig. 4:**
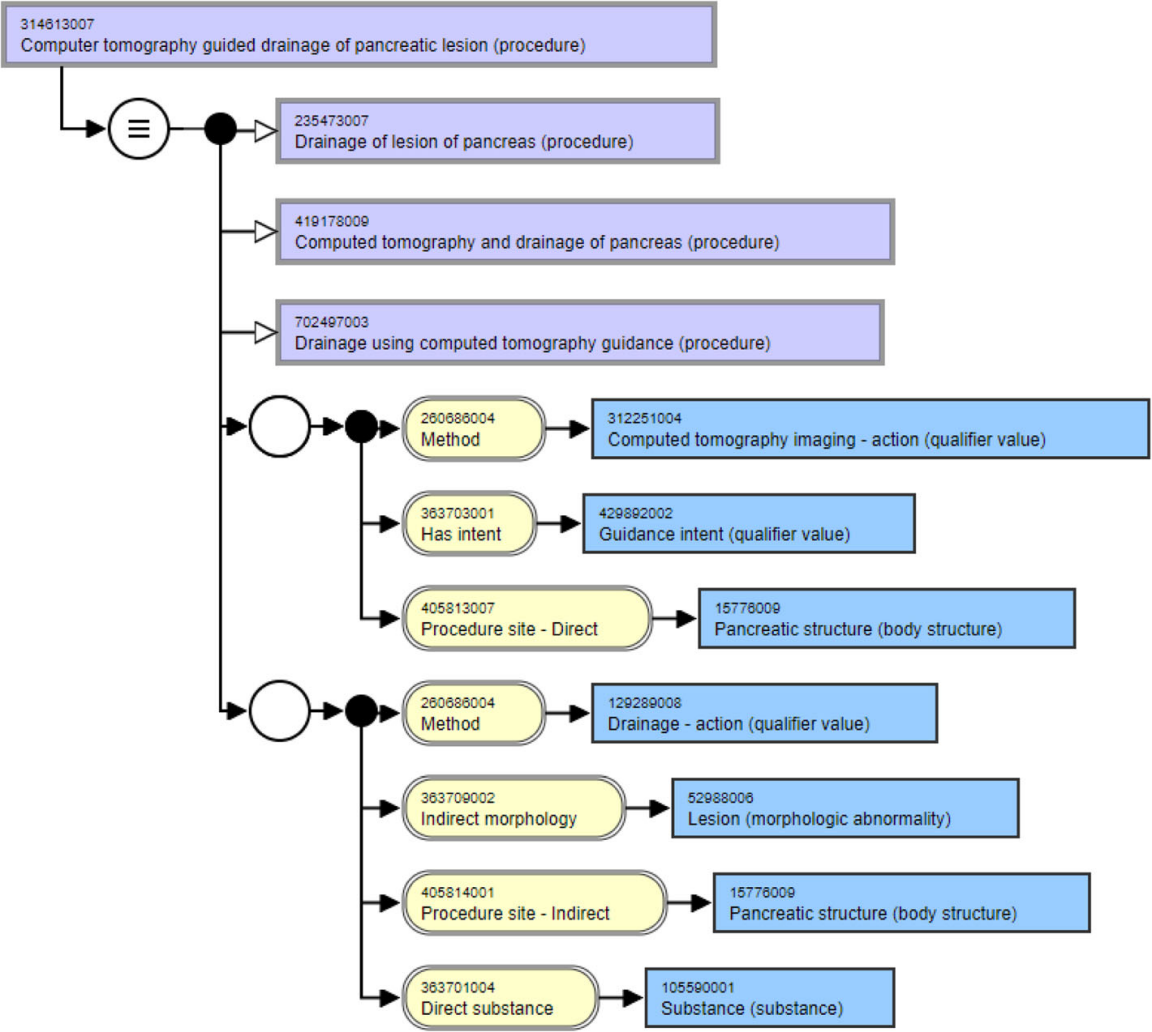
Structural modeling of concept with Concept ID 314613007

Consider the two concepts discussed in Table 2. While the two concepts are lexically similar based on their synonyms as shown in Table 3, they, however differ in their structural modeling as shown in Fig. 3 and Fig. 4. First, let’s consider their hierarchical relationships. While the three parents of the pancreatic concept are defined at a more granular level, the pelvis concept is modeled using more general parent concepts. It is suggested that the parent concepts of pelvis concept be made more specific in line with the pancreatic concept. The concept “Drainage using computed tomography guidance (procedure)” can be added as a parent to the pelvis concept similar to the pancreatic concept. The parent concepts “Computerized axial tomography (procedure)” and “Drainage procedure (procedure)” of the pelvis concept can be replaced with the more specific “Computed tomography and drainage of pelvis (procedure)” similar to the pancreatic concept.

Now, consider the attributes and relationship groups of these two concepts. While both these concepts have two relationship groups each, there are inconsistencies with the number of relationships in the groups. While the first group of the pelvis concept has the attribute “Procedure site - Indirect”, the attribute is “Procedure site -Direct” for the pancreatic concept. Besides, this first relationship group in the pancreatic concept has an additional attribute “Has intent” with the target “Guidance intent” which is missing in the pelvis concept. Similarly, the second group of the pancreatic concept has an attribute “Indirect morphology” with the target value “Lesion” which is missing from the second group of the pelvis concept. This example clearly illustrates the value of the method and how it can be used to identify inconsistencies in the modeling of the concepts when looked at in the context of lexically similar concepts.

One of the benefits of using a standardized terminology such as SNOMED CT in EHRs is the generation of computable data that can be used to query the system for analytics, research and clinical decision support. The methodology described in this study helps improve analytics and decision support capabilities of SNOMED CT by identifying and helping minimize inconsistencies in the concept modeling.

As an example, consider the similarity set in Table 8 consisting of two concepts. While the first stage concept has a parent “First stage of staged operation (procedure)”, the second stage concept is missing a similar parent. The January 2018 release of SNOMED CT has a concept “Subsequent stage of staged operation (procedure)” which can be added as a parent of this concept to make the modeling of these similar concepts consistent. Without this relationship, a query on patients with “Subsequent stage of staged operation (procedure)” would not return patients encoded with “Gross operation repair of omphalocele, second stage (procedure)”.

**Table 8:** Similarity set with concepts having inconsistent hierarchical relationship

Gross operation repair of omphalocele, first stage (procedure)	
Gross operation repair of omphalocele, second stage (procedure)	

As a second example, consider the similarity set consisting of two concepts as shown in Table 9. While the referral concept has an attribute “Method” with target value “Referral - action”, the discharge concept does not have any attribute. Adding the attribute “Method” with target value “Discharge - action” will add consistency to the modeling of these similar concepts. Besides, with this change, any query on “Discharge - action” will show patients who are coded with “Discharge from young disabled service (procedure)” in the system.

**Table 9:** Similarity set with concepts having inconsistent attributes

Referral to young disabled service (procedure)	
Discharge from young disabled service (procedure)	

There are certain limitations of the described method. The method only takes into account 36% of the concepts from the Procedure hierarchy. The rest 64% of the concepts in the hierarchy do not form sets and are not evaluated for inconsistent modeling. While on one hand, this is similar to most methodologies where “one size fits all” may not apply to auditing of biomedical terminologies, on the other hand, the described method uses multiple structural indicators such as hierarchical relationships, attributes and relationship groups and is able to cover over a third of the concepts in the Procedure hierarchy. Future plan involves looking into other structural indicators that can be used to identify inconsistencies in the modeling of the concepts.

## Conclusion

With the rising adoption of SNOMED CT to record clinical information in Electronic Health Records as well as its use in clinical decision support systems, it is imperative that SNOMED CT meet certain quality standards. The evaluation of Procedure hierarchy in this study shows that inconsistencies do exist in SNOMED CT. The methodology discussed, and its assessment demonstrates the value of this technique that can augment SNOMED International’s own quality assurance efforts to minimize inconsistencies in SNOMED CT.

## Abbreviations

CTV3: Clinical Terms Version 3
EHR: Electronic Health Record
HITECH: Health Information Technology for Economic and Clinical Health
SNOMED CT: SNOMED Clinical Terms
VA/KP: Veteran Administration/ Kaiser Permanente
